# Clinical Features of HBV‐Related HCC in Long‐Term NAs‐Treated Versus Untreated Patients

**DOI:** 10.1111/jcmm.70717

**Published:** 2025-07-13

**Authors:** Yuyu Ye, Yeqiong Zhang, Yunming Tang, Ming Liu, Shibin Xie, Ying Liu

**Affiliations:** ^1^ Department of Infectious Diseases The Third Affiliated Hospital of Sun Yat‐Sen University Guangzhou China; ^2^ Department of Infectious Diseases Hospital of Hainan Province Haikou China

**Keywords:** chronic hepatitis B, hepatocellular carcinoma, nucleos(t)ide analogue(s)

## Abstract

Patients who receive long‐term anti‐hepatitis B virus (HBV) treatment with nucleos(t)ide analogues (NAs) are still at risk for primary hepatocellular carcinoma (HCC). Aim The purpose of this study was to compare clinical features of HBV‐related HCC in NA‐treated vs. untreated patients. The records of patients who were diagnosed with HCC for the first time at the Third Affiliated Hospital of Sun Yat‐sen University (Guangzhou, China) between January 1, 2019 and September 31, 2024 were retrospectively reviewed. Patients with chronic HBV (CHB)‐related HCC were grouped into the NA‐treated group and untreated group. A total of 562 patients with CHB‐related HCC were identified and divided into the NA treatment group (*n* = 146) and the untreated group (*n* = 416). Patient age was similar between the groups (50.9 ± 10.1 vs. 52.5 ± 12.1 years, *p* > 0.05). HBV DNA level, alanine aminotransferase (ALT) level and gamma glutamyl transpeptidase (GGT) level were significantly lower in the NA group (all, *p* < 0.001). Alpha fetoprotein (AFP) level was significantly higher in the untreated group (*p* < 0.05). However, the HBeAg positive rate was significantly greater in the NA group (27.4% vs. 17.5%, *p* < 0.05), and79.5% (116/146) of HCCs occurred in the first 5 years of NA treatment. Blood biochemical indexes and AFP values of patients who develop CHB‐related HCC after NA treatment are usually normal. However, the high HBeAg seropositive rate in patients treated with NAs requires attention.

## 1 Introduction

Hepatitis B virus (HBV) infection remains a significant public health issue, in part due to its severe complications. Worldwide, 240–300 million people are chronically infected with the Hepatitis B virus (CHB), and 75% of these persons live in Asia [1]. CHB infection is responsible for about 30% of cirrhosis cases and 50% of newly diagnosed hepatocellular carcinoma (HCC) cases in endemic areas each year [2]. HCC is a highly aggressive tumour that is the third global cause of cancer‐related deaths [3, 4]. Approximately 50% of HCC cases are in China, and the primary reason is HBV infection. Therefore, the prevention and long‐term control of CHB complications are of great importance.

Since lamivudine was introduced for the treatment of hepatitis B in 1998, nucleos(t)ide analogues (NAs) have become the standard treatment due to their tremendous anti‐viral effect [5, 6]. Studies have shown that NAs can delay the onset of HCC and reduce the rate of other adverse outcomes, such as hepatic fibrosis and cirrhosis [7, 8, 9]. Moreover, NAs have been shown to improve survival and decrease recurrences in HCC patients after a curative resection [10, 11]. Long‐term NA therapy is recommended by professional organisation guidelines and expert consensus [12]. Nevertheless, a large number of patients with HBV develop HCC after long‐term NA treatment [13]. The underlying mechanisms for this remain unclear. There have been few studies that have examined the clinical features of patients with CHB who developed HCC after long‐term NA treatment [14, 15].

Increased serum HBV viral load is a well‐known risk factor for the development of HCC [16]. Other risk factors include age, sex, family history, low platelet level, cirrhosis, alcoholic abuse and comorbidities such as diabetes and hypertension [17, 18, 19]. While these factors are incorporated into many models to determine which patients are at high risk of developing HCC, the underlying clinical significance of these factors remains to be studied.

In China, since entecavir and tenofovir were included in the National Reimbursement Drug List (NRDL) in 2017, the accessibility of nucleoside analogues (NAs) has significantly improved. The price of entecavir has dropped by over 90%, from approximately 1500 yuan per month to 10 yuan per month, greatly enhancing affordability. However, due to the uneven distribution of medical resources and the limited awareness of primary care physicians, disparities still exist in rural areas. A 2019 meta‐analysis indicated that only 55% of eligible CHB patients in urban tertiary hospitals received antiviral therapy, compared to 32% in rural areas [20]. Thus, the purpose of this study was to compare the clinical features of HBV‐related HCC between patients treated long‐term with NAs and patients who did not receive any anti‐HBV treatment. Differences between these groups of patients may assist in identifying those at high risk for HCC, and allow interventions to reduce the risk.

## 2 Methods

### 2.1 Patients and Study Design

This retrospective study reviewed medical records of patients diagnosed with HCC at the Third Affiliated Hospital of Sun Yat‐sen University from January 1, 2019, to September 31, 2024.

### 2.2 Eligibility Criteria for NA Treatment

According to the Chinese Guidelines for Prevention and Treatment of Chronic Hepatitis B (2019):

(1) HBeAg‐positive patients with viral replication and elevated ALT; (2) Cirrhosis patients with high levels of HBV DNA; (3) Patients receiving treatment that may cause HBV reactivation, such as chemotherapy or immunosuppressive agents; (4) Patients with HBV infection after liver transplantation; (5) Patients with HBV‐related liver failure or HCC.

#### 2.2.1 Inclusion Criteria

(1) Diagnosis of HBV‐related HCC; (2) Complete clinical and laboratory data at the time of HCC diagnosis.

#### 2.2.2 Exclusion Criteria

(1) Extra‐hepatic malignancy; (2) Incomplete data; (3) Received interferon for > 12 weeks; (4) NA treatment for < 12 weeks during or before the observation period; (5) Co‐infected with hepatitis C virus (HCV) or HIV.

Patients were divided into two groups:

*NA‐treated group:* Received NA treatment (lamivudine, adefovir, telbivudine and entecavir) for at least 48 weeks.

*Untreated group:* Received NA treatment for < 24 weeks or no treatment.

The diagnosis of HCC was based on clinical, serological, radiological and histological evidence. The diagnostic criteria were consistent with those published in the European Association for the Study of the Liver (EASL) Clinical Practice Guidelines: Management of Hepatocellular Carcinoma. The Barcelona Clinic Liver Cancer (BCLC) score was calculated for each patient at the time of diagnosis to determine prognosis and optimise treatment.

Patient demographic data, the NA used and number of treatment cycles, time of HBV diagnosis, and family history of HBV and HCC used in the analysis were those obtained at the time of HCC diagnosis. Cirrhosis was assessed by liver biopsy or liver imaging (abdominal ultrasonography, computed tomography [CT] or magnetic resonance imaging [MRI]), which may show indirect manifestations of cirrhosis including portal hypertension, oesophageal‐gastric varices, splenomegaly and ascites. Data regarding alcoholic abuse and comorbidities such as diabetes and hypertension were also collected.

Laboratory data collected at the time of HCC diagnosis included biochemical indexes of liver function (alanine aminotransferase [ALT], gamma glutamyl transpeptidase [GGT] and albumin), HBV‐related parameters (hepatitis B surface antigen [HBsAg], hepatitis B surface antibody [HBsAb], hepatitis B e antigen [HBeAg], hepatitis B e antibody [HBeAb], hepatitis B core antibody [HBcAb], HBV DNA titre) and HCC indexes (alpha fetoprotein [AFP]) as well as tissue pathological examination and liver imaging results. At the same time, cirrhosis severity was classified using the Child‐Pugh score based on the following parameters:Albumin (g/dL), Total bilirubin (mg/dL), INR (international normalised ratio), Ascites (none, mild, moderate–severe), Hepatic encephalopathy (none, grade 1–2, grade 3–4). Scores were categorised as Class A (5–6 points), Class B (7–9 points) or Class C (10–15 points).

### 2.3 Statistical Analysis

Student's *t* test was used to compare continuous variables with normal distributions. Variables with a non‐normal distribution were compared by the Wilcoxon signed rank test. Chi‐square or Fisher's exact test was used to compare categorical variables. Continuous variables with normal distributions were described by mean ± standard deviation. Non‐normally distributed continuous variables were described by median (range). SPSS version 19.0 software was used to perform the statistical analyses, and a value of *p* < 0.05 was considered to indicate statistical significance. Figures were created using Graphpad Prism 7 software.

## 3 Results

### 3.1 Patient Characteristics

**FIGURE 1:**
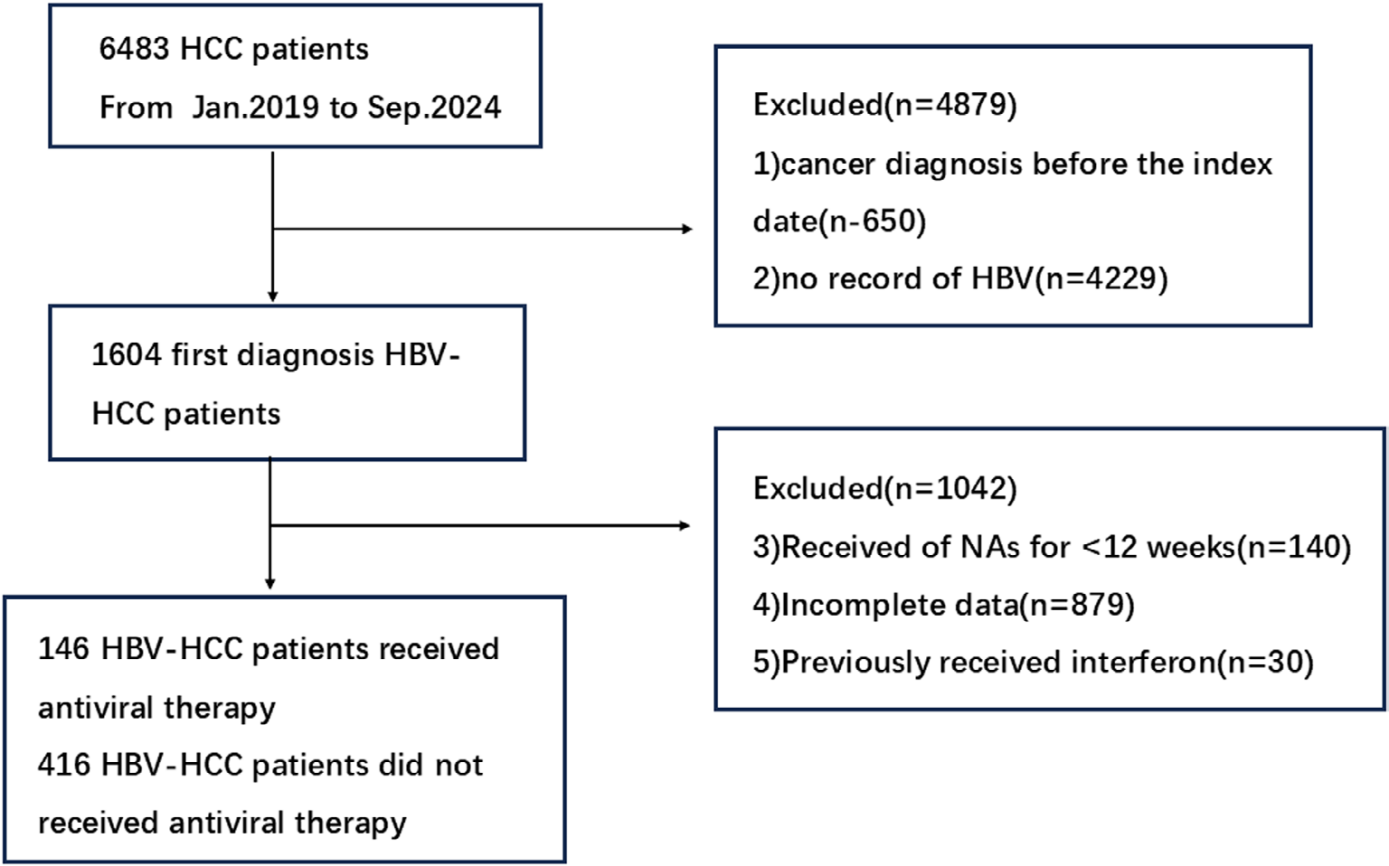
Flow diagram of patient inclusion. HBV, Hepatitis B virus; HCC, hepatocellular carcinoma.

The review of the medical records identified 562 patients with HBV‐related HCC that were eligible for inclusion in the analysis (Figure 1). There were 505 males and 53 females, with an age range from 30 to 86 years. Approximately 7.8% of the patients had a family history of HCC, and approximately 80% (451/562) had cirrhosis. It is noteworthy that the HBV viral load of 52% (295/562) of the patients was > 2000 IU/mL (Table 1).

**TABLE 1:** Characteristics of patients in NAs group and UT group.

	NAs group (*n* = 146)	UT group (*n* = 416)	*χ* ^2^	*p*
Age (year), Mn (SD)	50.9 (10.1)	52.5 (12.1)	1.401^a^	0.162
Gender, *n* (%)				
Male	131 (89.7)	378 (90.9)	0.550	0.458
Female	15 (10.3)	38 (9.1)		
HCC family history, *n* (%)				
No	130 (89.0)	388 (93.3)	2.844	0.092
Yes	16 (11.0)	28 (6.7)		
Cirrhosis^b^, *n* (%)				
No	18 (12.3)	93 (22.4)	7.439	0.024
Yes	128 (87.7)	323 (77.6)		
Child‐Pugh class, *n* (%)				
A	85 (58.2%)	160 (38.5%)	18.72	0.000
B	45 (30.8%)	180 (43.3%)		
C	16 (11.0%)	76 (18.2%)		
HBV DNA (IU/mL), *n* (%)				
< 500	121 (82.9)	66 (15.9)	218.393	0.000
500 ~ 2000	8 (5.5)	72 (17.3)		
≥ 2000	17 (11.6)	278 (66.8)		
HBeAg positive, *n* (%)	40 (27.4)	73 (17.5)	6.513	0.039
Alcoholic liver, *n* (%)	14 (9.6)	58 (13.9)	1.933	0.164
Diabetes, *n* (%)	16 (11.0)	28 (6.7)	2.572	0.109
ALT (U/L), *n* (%)				
< 40	107 (73.3)	141 (33.9)	269.554	0.000
40 ~ 120	31 (21.2)	172 (41.3)		
≥ 120	8 (5.5)	103 (24.8)		
GGT (U/L), Mn	87.5	137.0	6.464^c^	0.000
AFP (ng/mL), *n* (%)				
< 20	53 (36.3)	104 (25.0)	35.164	0.000
20 ~ 200	44 (30.1)	82 (19.7)		
200 ~ 400	19 (13.0)	32 (7.7)		
≥ 400	30 (20.5)	198 (47.6)		
BCLC stage, *n* (%)				
Stage 0 ~ Stage B	79 (54.1)	116 (27.9)	43.044	0.000
Stage C ~ Stage D	67 (45.9)	300 (72.1)		
Lesions Dmax (mm), Mn	37.2	72.0	7.749^c^	0.000
Number of lesions, *n* (%)				
1	98 (67.1)	136 (32.7)	56.482	0.000
≥ 2	48 (32.9)	280 (67.3)		
Vascular invasion, *n* (%)	36 (24.7)	251 (60.3)	53.309	0.000

Abbreviations: AFP, alpha fetoprotein; ALT, alanine aminotransferase; BCLC, Barcelona Clinic Liver Cancer; Dmax, Maximum diameter; DNA, deoxyribonucleic acid; GGT, gamma glutamyl transpeptidase; HBeAg, hepatitis B e antigen; HBV, hepatitis B virus; HCC, hepatocellular carcinoma; HCV, hepatitis C virus; Mn, mean; NAs Group, NAs treated group; SD, standard deviation; UT Group, untreated group.

^a^ Data presented by *t* values.

^b^ Cirrhosis was diagnosed by liver biopsy and/or clinical evidence.

^c^ Data presented by *Z* values.

### 3.2 Characteristics Treated and Untreated Patients

Of the562 patients, 146 received NA treatment and 416 did not receive NA treatment. The characteristics of the two groups are summarised in Table 1. The two groups were similar with respect to age, sex distribution and family history of HCC (all, *p* > 0.05). The proportion of patients with cirrhosis in the NA treatment group was significantly higher than in the untreated group (87.7% vs. 77.6%, *p* = 0.024), while the mean GGT level in the untreated group was significantly greater than in the treatment group (137.0 vs. 87.5 U/L, *p* < 0.001). The proportion of Child‐Pugh grade C in the untreated group was higher (18.2% vs. 11.0%, *p* < 0.05), reflecting that untreated patients were more likely to progress to end‐stage liver cirrhosis. Most patients in the NA treatment group had ALT values below 40 U/L (107/146, 73.3%), which is often considered a normal value. Significantly more patients in the untreated group had an AFP level ≥ 400 ng/mL (47.6% vs. 20.5%, *p* < 0.01). Approximately 67% of patients in the untreated group had a HBV DNA load ≥ 2000 IU/mL, as compared to 11.6% in the treatment group (*p* < 0.001). The majority of patients in the NA treatment group had a HBV DNA titre < 500 IU/mL. Notably, the HBeAg positive rate was significantly greater in the NA treatment group than in the untreated group (27.4% vs. 17.5%, *p* = 0.039).

**FIGURE 2:**
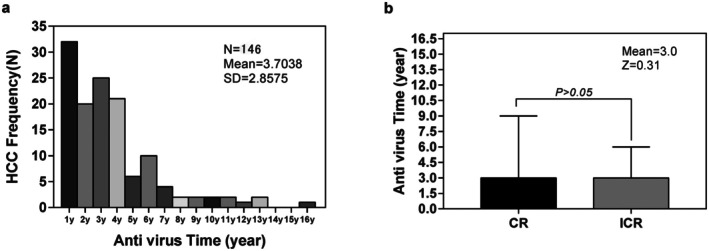
(a) Durations of NA treatments. (b) Comparison of the duration of NA treatment between complete response patients and incomplete response patients. Treatment durations were similar between the groups. CR, complete virological response; ICR, incomplete virological response.

In order to better understand differences in the cancer characteristics of the two groups, the HCC characteristics were compared. More than half of patients in the untreated group had an advanced BCLC score when HCC was diagnosed (300/416, 72.1%), and poor prognosis based on the number of lesions, size of lesions and vascular invasion (Table 1). In the NA treatment group, the median time of HCC diagnosis was 3 years after beginning NA treatment (mean 3.7 ± 2.9 years), and 80.0% of HCCs occurred within the first 5 years after beginning NA treatment. There were 8 patients who received NA treatment for more than 10 years, and all of them achieved a complete virological response. The distribution of the duration of NA treatment is shown in Figure 2.

### 3.3 Clinical Features of the NA Treatment Group

Of the 146 patients in the NA treatment group, there were 131 males and 15 females with an age range of 30–61 years. At the time of HCC diagnosis, 20 patients had achieved HBeAg seronegative conversion, and all of the patients had a complete virological response.

Of the 146 patients, 126 patients received a single NA treatment regimen, including entecavir (*n* = 44), lamivudine (*n* = 35), adefovir dipivoxil (*n* = 25), tenofovir disoproxil fumarate (*n* = 16) and telbivudine (*n* = 6). The remaining patients received a combination of drugs (*n* = 20). Lamivudine and entecavir were the 2 most common monotherapies (35/146, 44/146), while lamivudine and adefovir dipivoxil were the most common combination therapy (14/20). Statistical analysis showed that there were no significant differences in AFP, GGT, tumour stage, lesion diameter, and lesion number distribution between the different treatment regimens (all, *p* > 0.05).

### 3.4 Characteristics of HCC Patients With Different Virological Responses

Patients in the NA treatment groups were categorised into those that had a complete virological response (*n* = 121, CR group) and those that had an incomplete virological response (*n* = 25, ICR group). Complete Virological Response (CR) means sustained HBV DNA suppression below the lower limit of detection (< 20 IU/mL) for ≥ 12 months, accompanied by HBeAg seroconversion (if HBeAg‐positive at baseline). While incomplete virological response (ICR) means failure to achieve HBV DNA < 20 IU/mL or virological breakthrough (≥ 1 log10 IU/mL increase in HBV DNA after initial suppression). The two groups were similar with respect to age, anti‐HBV treatment duration, sex distribution, HCC family history, cirrhosis, alcoholic liver disease and diabetes (all, *p* > 0.05). The HBeAg seropositive rate of the CR group was significantly lower than that of the ICR group (24% vs. 44%, *p* < 0.05) (Table 2).

**TABLE 2:** Characteristics of patients with different virological responses.

	CR group (*n* = 121)	ICR group (*n* = 25)	*X* ^*2*^	*p*
Age (year), Mn (SD)	51.4 (10.3)	47.9 (9.5)	1.316^a^	0.190
Anti‐HBV time (year), Mn	3.7	3.8	0.534^b^	0.594
Gender, *n* (%)				
Male	112 (92.6)	19 (76.0)	2.289	0.130
Female	9 (7.4)	6 (24.0)		
HCC family history, *n* (%)				
No	108 (89.3)	22 (88.0)	0.034	0.855
Yes	13 (10.7)	3 (12.0)		
Cirrhosis^c^, *n* (%)				
No	14 (11.6)	4 (16.0)	0.744	0.388
Compensated	64 (52.9)	13 (52.0)		
Decompensated	43 (35.5)	8 (32.0)		
Child‐Pugh Class, *n* (%)				
A	60 (49.6%)	14 (56.0%)	2.69	0.26
B	40 (33.1%)	10 (40.0%)		
C	21 (17.4%)	1 (4.0%)		
Alcoholic liver, *n* (%)	13 (10.7)	1 (4.0)	1.643	0.200
Diabetes, *n* (%)	15 (12.4)	1 (4.0)	1.079	0.299
HBeAg positive, *n* (%)	29 (24.0)	11 (44.0)	6.007	**0.014**
ALT (U/L), *n* (%)				
< 40	95 (78.5)	12 (48.0)	9.579	**0.002**
40–120	22 (18.2)	9 (36.0)		
≥ 120	4 (3.3)	4 (16)		
GGT (U/L), Mn	82.0	122.4	1.792^b^	0.076
AFP (ng/mL), *n* (%)				
< 20	46 (38.2)	7 (28.0)	5.607	**0.018**
20–200	40 (33.1)	4 (16.0)		
200–400	14 (11.6)	5 (20.0)		
≥ 400	21 (17.4)	9 (36.0)		
BCLC stage, *n* (%)				
Stage 0 ~ Stage B	69 (57.0)	10 (40.0)	7.700	0.053
Stage C ~ Stage D	52 (43.0)	15 (60.0)		
Lesions Dmax (mm), Mn	35.0	46.0	1.545^b^	0.125
Number of lesions, *n* (%)				
1	82 (67.8)	16 (64.0)	1.871	0.171
≥ 2	39 (32.2)	9 (36.0)		
Vascular invasion, *n* (%)	26 (21.5)	10 (40.0)	2.664	0.103

*Note:* Bold values denote statistically significant differences between groups (*P* < 0.05).

Abbreviations: AFP, alpha fetoprotein; ALT, alanine aminotransferase; BCLC, Barcelona Clinic Liver Cancer; CR Group, complete virological response group, HBV DNA was detected lower than the lower limit by PCR after antiviral treatment; Dmax, maximum diameter; GGT, gamma glutamyl transpeptidase; HBeAg, hepatitis B e antigen; HBV, hepatitis B virus; HCC, hepatocellular carcinoma; ICR Group, incomplete virological response group, HBV DNA decreased after antiviral therapy by PCR, but failed to reach below the lower limit of detection; Mn, mean; SD, standard deviation.

^a^ Data presented by *t* values.

^b^ Data presented by *Z* values.

^c^ Cirrhosis was diagnosed by liver biopsy and/or clinical evidence.

HCC patients in the CR group had significantly lower ALT and AFP levels than those in the ICR group (both, *p* < 0.05) (Table 2). The proportion of patients in the ICR group with an AFP level > 400 ng/mL was significantly higher than in the CR group (36% vs. 17.4%, *p* < 0.05), as was the proportion with an ALT level > 120 U/L (16% vs. 3.3%, *p* < 0.05). However, there were no significant differences in BCLC stage and the number of lesions between the two groups (both, *p* > 0.05). Lesion size was smaller, and the frequency of metastasis was lower in the CR group (both, *p* < 0.05), underscoring the importance of achieving CR for HCC risk mitigation.

### 3.5 Clinical Features of the Untreated Group

The 416 patients who did not receive treatment were categorised into those who were HBV DNA negative (*n* = 66) and HBV DNA positive (*n* = 350). The proportion of patients with decompensated cirrhosis was significantly greater in the HBV DNA positive group (38.6% vs. 16.7%, *p* = 0.003). In addition, the HBeAg positive rate was significantly higher in the HBV DNA positive group (19.4% vs. 7.6%, *p* < 0.05) (Table 3). At the time of HCC diagnosis, 13 cases of HBeAg seronegative conversion were observed; 2 in the HBV DNA negative group and 11 in the HBV DNA positive group. Thus, the seropositive HBsAg rate was 93.3% (388/416) and the HBeAg rate was 17.5% (73/416).

**TABLE 3:** Characteristics of patients with different seropositive results of viral DNA in the UT group.

	Non‐antiviral DNA negative group (*n* = 66)	Non‐antiviral DNA positive group (*n* = 350)	*X* ^*2*^	*p*
Age (year), Mn (SD)	55.1 (11.5)	52.0 (12.2)	1.958^a^	0.051
Gender, *n* (%)				
Male	59 (89.4)	319 (91.1)	0.205	0.817
Female	7 (10.6)	31 (8.9)		
Family history, *n* (%)				
No	64 (97.0)	324 (92.6)	1.873	0.355
HCC history	1 (1.5)	22 (6.5)		
Other tumours	1 (1.5)	3 (0.9)		
Cirrhosis^b^, *n* (%)				
No	18 (27.3)	75 (21.4)	11.806	**0.003**
Compensated	37 (56.0)	140 (40.0)		
Decompensated	11 (16.7)	135 (38.6)		
Child‐Pugh class, *n* (%)				
A	35 (53.0%)	125 (35.7%)	7.31	0.026
B	22 (33.3%)	158 (45.1%)		
C	9 (13.6%)	67 (19.1%)		
Alcoholic liver, *n* (%)	9 (13.6)	49 (14.0)	0.006	1.000
Diabetes, *n* (%)	2 (3.0)	26 (7.4)	1.711	0.283
HBeAg positive, *n* (%)	5 (7.6)	68 (19.4)	5.770	**0.016**
ALT (U/L), *n* (%)				
< 40	36 (54.5)	105 (30.0)	15.109	**0.001**
40–120	20 (30.3)	152 (43.4)		
≥ 120	10 (15.2)	93 (26.6)		
GGT (U/L), *n* (%)	86.0	149.0	3.531^c^	**0.000**
AFP (ng/mL), *n* (%)				
< 20	21 (31.8)	76 (21.7)	2.958	0.400
20–200	11 (16.7)	71 (20.3)		
200–400	5 (7.6)	27 (7.7)		
≥ 400	29 (43.9)	176 (50.3)		
BCLC stage, *n* (%)				
Stage 0 ~ Stage B	27 (40.9)	89 (25.4)	6.618	**0.012**
Stage C ~ Stage D	39 (59.1)	261 (74.6)		
Lesions Dmax (mm), Mn	78.0	69.5	0.570^c^	0.568
Number of lesions, *n* (%)				
1	30 (45.5)	106 (30.3)	5.132	**0.032**
≥ 2	36 (54.5)	244 (69.7)		
Vascular invasion, *n* (%)	33 (50.0)	218 (62.3)	3.608	0.074

*Note:* Bold values denote statistically significant differences between groups (*P* < 0.05).

Abbreviations: AFP, alpha fetoprotein; ALT, alanine aminotransferase; BCLC, Barcelona Clinic Liver Cancer; Dmax, maximum diameter; GGT, gamma glutamyl transpeptidase; HBeAg, hepatitis B e antigen; HBsAg, hepatitis B surface antigen; HBV, hepatitis B virus; HCC, hepatocellular carcinoma; Mn, mean; SD, standard deviation.

^a^ Data presented by *t* values.

^b^ Cirrhosis was diagnosed by liver biopsy and/or clinical evidence.

^c^ Data presented by *Z* values.

In the HBV DNA negative group ALT and GGT levels were significantly lower than in the positive group (< 40 U/L, 54.5% vs., 30.0%; 86.0 vs., 149.0 U/L, respectively, both, *p* < 0.01). The frequency of BCLC score of 0 to B was significantly higher in the negative group, suggesting an earlier tumour stage (40.9% vs. 25.4%, *p* < 0.05). However, AFP level, lesion size, lesion number, and the degree of vascular infiltration were similar between the two groups at the time of HCC diagnosis (all, *p* > 0.05) (Table 3).

### 3.6 Clinical Features of the NA Complete Virological Response Group and Untreated Patients With Negative HBV DNA

A further comparison was done between patients treated with NAs who had a complete virological response and patients who were not treated and were HBV DNA negative. The two groups were similar with respect to sex distribution, family history and alcoholic liver disease (all, *p* > 0.05). However, the mean age of patients in the complete virological response group was significantly lower (51.4 ± 10.3 vs. 55.1 ± 11.5 years, *p* < 0.05), but there was a greater proportion of patients with decompensated cirrhosis (35.5% vs. 16.7%, *p* < 0.01) and diabetes (12.4% vs. 3.0%, *p* < 0.05). Furthermore, the HBeAg positive rate was significantly higher in the complete response group (24.0% vs. 7.6%, *p* < 0.01).

Although ALT levels in both groups were within the normal range (< 40 U/L), GGT levels in the non‐treatment DNA negative group were higher (86.0 vs. 82 U/L, *p* = 0.001). In the complete virological response group, the frequency of patients with AFP levels < 20 ng/mL was significantly higher (38.2% vs. 31.8%, *p* < 0.001), while in the untreated DNA negative group the frequency of patients with AFP levels ≥ 400 ng/mL was significantly higher (43.9% vs. 17.4%, *p* < 0.001). Other indicators, such as tumour stage, size, number of lesions and vascular invasion, all showed the characteristics of earlier stage, smaller lesions, fewer lesions and invasiveness in CR group (*p* < 0.01) (Table 4).

**TABLE 4:** Characteristics of patients in complete virological response group and non‐antiviral DNA negative group.

	Complete virological response group (*n* = 121)	Non‐antiviral DNA negative group (*n* = 66)	*X* ^*2*^	*p*
Age (year), Mn (SD)	51.4 (10.3)	55.1 (11.5)	2.304^a^	**0.022**
Gender, *n* (%)				
Male	112 (92.6)	59 (89.4)	0.001	0.973
Female	9 (7.4)	7 (10.6)		
Family history, *n* (%)				
No	107 (88.4)	64 (97.0)	4.715	0.030
HCC history	13 (10.7)	1 (1.5)		
Other tumour	1 (0.8)	1 (1.5)		
Cirrhosis^b^, *n* (%)				
No	14 (11.6)	18 (27.3)	11.499	**0.003**
Compensated	64 (52.9)	37 (56.0)		
Decompensated	43 (35.5)	11 (16.7)		
Alcoholic liver, *n* (%)	13 (10.7)	9 (13.6)	0.344	0.557
Diabetes, *n* (%)	15 (12.4)	2 (3.0)	4.533	**0.033**
HBsAg positive, *n* (%)	120 (99.2)	60 (90.9)	7.866	**0.005**
HBeAg positive, *n* (%)	29 (24.0)	5 (7.6)	6.608	**0.010**
ALT (U/L), *n* (%)				
< 40	95 (78.5)	36 (54.5)	12.642	**0.000**
40–120	22 (18.2)	20 (30.3)		
≥ 120	4 (3.3)	10 (15.2)		
GGT (U/L), Mn	82.0	86.0	3.544^c^	**0.001**
AFP (ng/mL), *n* (%)				
< 20	46 (38.2)	21 (31.8)	16.623	**0.001**
20–200	40 (33.1)	11 (16.7)		
200–400	14 (11.6)	5 (7.6)		
≥ 400	21 (17.4)	29 (43.9)		
BCLC stage, *n* (%)				
Stage 0 ~ Stage B	69 (57.0)	27 (40.9)	3.150	0.076
Stage C ~ Stage D	52 (43.0)	39 (59.1)		
Lesions Dmax (mm), Mn	35.0	78.0	8.145^c^	**0.000**
Number of lesions, *n* (%)				
1	82 (67.8)	30 (45.5)	15.627	**0.000**
≥ 2	39 (32.s2)	36 (54.5)		
Vascular invasion, *n* (%)	26 (21.5)	33 (50.0)	16.076	**0.000**

*Note:* Bold values denote statistically significant differences between groups (*P* < 0.05).

Abbreviations: AFP, alpha fetoprotein; ALT, alanine aminotransferase; BCLC, Barcelona Clinic Liver Cancer; Dmax, maximum diameter; GGT, gamma glutamyl transpeptidase; HBeAg, hepatitis B e antigen; HBsAg, hepatitis B surface antigen; HBV, hepatitis B virus; HCC, hepatocellular carcinoma; Mn, mean; SD, standard deviation.

^a^ Data presented by *t* values.

^b^ Cirrhosis was diagnosed by liver biopsy and/or clinical evidence.

^c^ Data presented by *Z* values.

## 4 Discussion

In this study, we compared the clinical characteristics of patients with HBV‐related HCC who either received NA treatment or did not receive treatment. Overall, the NA treatment group exhibited better indexes of liver function, lower HBV DNA titers, and an earlier HCC stage, all of which often indicate a favourable prognosis. In this study, a large proportion of patients in both groups had cirrhosis; however, the frequency of 77.6% in the untreated group deserves attention. A large part of the reason lies in the large population base, patients from different regions in south China, and different health care awareness. Liver cirrhosis has long been recognised as a risk factor for HCC in CHB patients [21]. Our data suggest that treatment with NAs after the onset of cirrhosis does not reduce the incidence or delay the development of HCC, which is consistent with the current mainstream view. A high viral load has been proven to be an important risk factor for the development of HCC [22, 23]. Although the level of HBV DNA in the NA treatment group was lower, it is accompanied by a high HBeAg seropositive rate, suggesting that long‐term suppression of the HBV may result in a higher mutation rate after reactivation.

The earlier tumour stage observed in the NA treatment group suggests that antiviral therapy may contribute to better monitoring and earlier detection of HCC. This may be because patients treated with NAs had better compliance with treatment and follow‐up, and thus HCC was detected at an earlier stage. Additionally, the improvement in liver function indices and suppression of HBV DNA levels in the NA group may delay HCC progression to more advanced stages. On the other hand, in untreated patients, HCC was detected when there were obvious clinical symptoms and thus the cancer was at a more advanced stage at diagnosis. Thus, this finding highlights the importance of routine surveillance even in patients with normalised liver function.

While the parameters studied were superior in the NA treatment group as compared to the untreated group, the age at which HCC was diagnosed was similar between the groups. This may be because the mean age at diagnosis was > 50 years in both groups, and the incidence of HCC increases after 50 years of age [24].

Treatment with NAs did not significantly reduce the risk of HCC as compared to no treatment, and we examined if this finding was related to cirrhosis. Treatment with NAs is recommended for patients with underlying cirrhosis, and studies have shown that it will slow progression of the disease [25]. The proportion of patients with cirrhosis was higher in the NA treatment group than in the untreated group, and most patients received NA treatment for up to 5 years, indicating that most of them received NA treatment after they were diagnosed with cirrhosis. In addition, due to the large number of patients, we cannot ensure that their regular medication, as well as subtle liver pathological changes in the course of medication, are covered up by NAs. During the natural process of HBV infection, the DNA double‐stranded gap is complemented to form covalently closed circle DNA (cccDNA) by DNA polymerase, and this is closely related to the occurrence of HCC [26, 27]. This residual cccDNA may contribute to the integration of HBV DNA into the host genome, promoting clonal expansion of hepatocytes and oncogenesis. While NA treatment effectively inhibits virus replication, it does not eradicate cccDNA, particularly in HBeAg‐positive patients with high levels of viral replication [28], which is the root cause of refractory hepatitis B, which increases the risk of HCC [29, 30]. This may be because of the exhaustion and dysfunction in T and B cells [31], but the specific mechanisms still need to be further studied. In a future study, we plan to follow younger patients without cirrhosis treated with NAs and determine if earlier NA treatment is associated with greater benefits.

Our findings are partially consistent with previous research, which has shown that NA therapy reduces but does not eliminate the risk of HCC. As observed in this study, long‐term NA treatment can delay HCC onset and reduce tumour burden, where NA‐treated patients had smaller tumours, fewer lesions, and lower rates of vascular invasion. But this finding does not seem to hold for patients over 50 years of age, those with cirrhosis, and those with HCV and HDV infection [32]. Similarly, in our study, the seropositive rate of HBeAg failed to reach an ideal level in the NAs group, even in patients who have achieved a complete virological response. Achieving the goal of undetectable HBV DNA and HBeAg seroconversion is far from a cure for CHB; the occurrence and development of CHB are more associated with immune status [33]. Furthermore, the high HBeAg positivity rate in our study aligns with reports suggesting that HBeAg positivity is associated with an increased risk of HCC, potentially due to ongoing viral replication and immune activation.

It is also noteworthy that the low AFP levels observed in a substantial proportion of NA‐treated patients highlight the limitations of AFP as a sole biomarker for HCC screening. This underscores the importance of incorporating regular imaging modalities, such as ultrasound, CT or MRI, into surveillance protocols for high‐risk populations, even in patients with normal liver function indices and low AFP levels. These findings illustrate the need for a more accurate and specific diagnostic method [34, 35], and the identification of additional biomarkers for liver cancer, such as circular RNAs [36]. Additionally, the development of risk stratification models incorporating clinical, biological and imaging data could help tailor surveillance strategies to individual patient profiles.

There are some limitations to this study that should be considered. It was a single‐centre, retrospective study with a limited sample size due to incomplete data. While we attempted to mitigate this limitation by including a relatively large sample size and diverse patient profiles, future multi‐centre studies are needed to validate and extend these findings across broader populations. In addition, patients may have recall bias, such as when they first began using NAs. To minimise these issues, we carefully screened and excluded patients with insufficient data, but the possibility of residual bias remains. Due to the large population base of patients, we cannot guarantee that patients are followed up regularly and whether NAs play a continuous role in their treatment. Some important research indicators and dynamic evolution data including the overall survival and NAs use post HCC treatment cannot be fully collected in the follow‐up process.

In conclusion, patients with CHB treated with long‐term NAs are still at risk of developing HCC, especially during the first 5 years of treatment, and patients who are cirrhotic and are HBeAg seropositive. Blood biochemical indices and AFP levels in patients treated with NAs are often normal, which makes detection of HCC more difficult. These findings underscore the need for continued vigilance and optimised surveillance strategies in this population. A deeper understanding of the mechanisms driving HCC in NA‐treated patients, along with the development of novel therapeutic and diagnostic tools, is critical to reducing the global burden of HBV‐related HCC.

## Author Contributions

**Yuyu Ye:** writing – original draft (equal). **Ying Liu:** project administration (equal), resources (equal). **Yeqiong Zhang:** data curation (equal), methodology (equal). **Yunming Tang:** writing – original draft (equal). **Ming Liu:** investigation (equal), methodology (equal). **Shibin Xie:** supervision (equal).

## Ethics Statement

This study complies with the Declaration of Helsinki and has been approved by the Human Ethics Committee, the Third Affiliated Hospital of Sun Yat‐sen University.

## Consent

The authors have nothing to report.

## Conflicts of Interest

The authors declare no conflicts of interest.

## Data Availability

The datasets generated during and/or analysed during the current study are available from the corresponding author on reasonable request.
